# Predictive genetic testing for the identification of high-risk groups: a simulation study on the impact of predictive ability

**DOI:** 10.1186/gm267

**Published:** 2011-07-28

**Authors:** Raluca Mihaescu, Ramal Moonesinghe, Muin J Khoury, A Cecile JW Janssens

**Affiliations:** 1 Department of Epidemiology, Erasmus University Medical Center, PO Box 2040, 3000 CA Rotterdam, The Netherlands; 2 Office of Minority Health and Health Disparities, Centers for Disease Control and Prevention, 1600 Clifton Road NE, Atlanta, GA 30341, USA; 3 Office of Public Health Genomics, Centers for Disease Control and Prevention, 1600 Clifton Road NE, Atlanta, GA 30341, USA

## Abstract

**Background:**

Genetic risk models could potentially be useful in identifying high-risk groups for the prevention of complex diseases. We investigated the performance of this risk stratification strategy by examining epidemiological parameters that impact the predictive ability of risk models.

**Methods:**

We assessed sensitivity, specificity, and positive and negative predictive value for all possible risk thresholds that can define high-risk groups and investigated how these measures depend on the frequency of disease in the population, the frequency of the high-risk group, and the discriminative accuracy of the risk model, as assessed by the area under the receiver-operating characteristic curve (AUC). In a simulation study, we modeled genetic risk scores of 50 genes with equal odds ratios and genotype frequencies, and varied the odds ratios and the disease frequency across scenarios. We also performed a simulation of age-related macular degeneration risk prediction based on published odds ratios and frequencies for six genetic risk variants.

**Results:**

We show that when the frequency of the high-risk group was lower than the disease frequency, positive predictive value increased with the AUC but sensitivity remained low. When the frequency of the high-risk group was higher than the disease frequency, sensitivity was high but positive predictive value remained low. When both frequencies were equal, both positive predictive value and sensitivity increased with increasing AUC, but higher AUC was needed to maximize both measures.

**Conclusions:**

The performance of risk stratification is strongly determined by the frequency of the high-risk group relative to the frequency of disease in the population. The identification of high-risk groups with appreciable combinations of sensitivity and positive predictive value requires higher AUC.

## Background

There is increasing interest in the potential use of testing multiple genetic variants for the prediction of common complex diseases such as type 2 diabetes, osteoporosis and cardiovascular disease, particularly because this could help targeting preventive and therapeutic interventions to individuals and groups with high genetic risk. While to date most genetic risk models show only modest predictive performance [1-7], improved prediction is expected when many new genetic risk factors are discovered in the coming years, both common and rare variants with intermediate to large effects on disease risk. Notwithstanding these anticipated discoveries, the predictive ability of genetic risk models for complex diseases is likely to remain modest because non-genetic risk factors have a substantial impact on disease risk as well [8,9].

Despite the modest predictive ability, some argue that genetic risk models can still be useful in health care and disease prevention to identify individuals at very high risk [10]. Preventive strategies can be targeted to individuals at very high risk even though this may only be a small subgroup [11,12]. The feasibility of this strategy will depend not solely on the predictive ability of the risk model, but also on the threshold level that is chosen. For certain diseases, well defined clinical cut-off values exist, such as the Framingham risk score for cardiovascular disease [13,14], but in most instances the relevant thresholds have not been determined. Risk thresholds are chosen on a cost-benefit analysis of false negative and false positive findings across all thresholds, and generally are a trade-off. High threshold values are needed to identify individuals with a high probability to develop future disease, but this may identify only a fraction of the patients, whereas lower thresholds will identify most individuals who will develop the disease but also classify many individuals wrongly at increased risk. Therefore, apart from the discriminative accuracy of the risk model, the threshold chosen has a major impact on the sensitivity, specificity, positive predictive value (PPV) and negative predictive value (NPV) when the risk model is used as a dichotomous test.

For single genetic tests, the relationship between the epidemiological assessment of the genetic association (for example, genotype frequency and odds ratio (OR)) and the predictive accuracy of the test (for example, sensitivity and PPV) have been described by simple arithmetic formulas [15]. These formulas show that the frequency of the risk variant relative to the frequency of disease determines whether the test will have high sensitivity or high PPV, and that both can be maximized only when genotype and disease frequencies are approximately equal. For instance, screening for a common disease using rare variants can detect only a few individuals at very high risk. Conversely, screening for a rare disease using common variants detects most individuals that will ultimately develop the disease at the cost of many false positive findings. It would be of interest to make use of the genomic era developments in this analysis by including multiple risk variants.

In this study, we examined the performance of risk stratification based on genetic risk models that include multiple variants simultaneously. We investigated sensitivity, specificity, PPV and NPV of genetic risk models along the range of threshold values that can be chosen to define high-risk groups. This detailed exploration of the interrelationships between sensitivity, PPV, prevalence of risk group and disease prevalence using genetic risk scores instead of single risk variants has not been reported before. We repeated the analyses for thresholds that define high-risk groups with a frequency lower, equal or higher than the disease frequency for increasing values of the area under the receiver operating characteristic curve (AUC). To address these objectives we used simulated data across a wide variety of ORs and frequencies for genetic variants. We also carried out an additional simulation based on published ORs and frequencies for six genetic polymorphisms predicting age-related macular degeneration (AMD) risk [16].

## Materials and methods

### Simulated data

For the construction of simulated data sets, we used a modeling procedure that has been described in detail elsewhere [8]. In short, the procedure creates a dataset in such a way that the frequencies and ORs of the risk genotypes and the disease risk match prespecified values. For simplicity, we assumed that each individual polymorphism had only two genotypes, one of which was associated with an increased risk of disease and the other with the referent or baseline risk. We assumed that genetic variants are inherited independently and that their joint effects follow a multiplicative risk model. And finally, we did not include gene-gene and gene-environment interactions in our analyses, which may further improve the predictive ability of genetic risk models. While these assumptions do impact the exact estimate of the AUC - for example, modeling interaction effects might give higher AUC - they do not affect the main aim of our paper, namely impact of a given AUC on the sensitivity, specificity, PPV and NPV for different thresholds of the genetic risk model. The population size was 10,000 individuals and the population disease risk was varied across scenarios (that is, 10% and 30%, respectively). We simulated 50 genetic risk factors, each having a risk genotype with a frequency of 30% and an OR that varied across scenarios (that is, 1.1, 1.5 and 2.0, respectively).

### Simulation study of age-related macular degeneration

We constructed a dataset using the disease risk from prevalence estimates in adults 40 years of age or over [17], and genotypic parameters from a published risk prediction model for AMD [16]. We used the same modeling procedure as in our main simulation study and a sample size of 10,000 individuals. The model included six genetic risk variants in the following genes or gene regions: *CFH *(rs1061170, rs1410996), *LOC387715 *(rs10490924), *C2 *(rs9332739), *CFB *(rs641153) and *C3 *(rs2230199). For each locus we considered the effect from the univariate logistic regression analysis with AMD as outcome variable and the genetic variants as predictor variables. For each locus the three genotypes were entered independently, with the exception of *C2 *and *CFB *for which the genotypes were grouped in two categories, one conferring an increased risk of disease. Additional file 1 shows genotype ORs and genotypic frequencies in controls. The prevalence of disease in the AMD simulation was 9% [17].

### Statistical analyses

In the main simulation study, we constructed a genetic risk score that was a simple count of the number of risk genotypes. Note that this score has perfect correlation with predicted risk because all variants have the same frequency of the risk genotype and the same OR. The disease risk increases with the number of risk genotypes in the genetic risk model. In the AMD simulation, we derived predicted risks using logistic regression analysis with genetic risk variants entered as categorical variables. High-risk groups were defined as all individuals with risk scores above a chosen threshold.

First, to evaluate the impact of genotype frequencies and ORs on the overall discriminative accuracy of genetic risk models, we assessed the AUC [18]. Next, to assess the predictive performance of genetic risk models for defining the high-risk group, we calculated the sensitivity, specificity, PPV and NPV for each possible threshold. The sensitivity is the percentage of individuals classified at high-risk among affected individuals and specificity is the percentage of individuals classified as not being at high-risk among unaffected individuals. PPV is the probability that individuals classified at high-risk will develop the disease, and NPV is the probability that individuals classified as not being at high-risk will remain free of disease. All measures are presented against cut-off values and the percentage of individuals at high-risk to examine the impact of the frequency of the high-risk group on the relationship between the sensitivity, specificity, PPV and NPV. Note that the frequency of the high-risk group defined by a certain threshold is different from the frequency of the risk genotype of each single genetic marker. Finally, to replicate the comparison between epidemiological assessment and predictive accuracy of the test [15], we assessed sensitivity, specificity, PPV and NPV for increasing AUC, in high-risk groups with a frequency lower, equal or higher than the disease risk. For this purpose, the threshold values were chosen such that the frequency of the high-risk groups was 5%, 30% or 50% as the disease risk was 30%. To achieve variation in AUC, we modeled 5 to 600 variants with OR of 1.1 and risk genotype frequency of 30%. Results are presented as means from 100 simulations. All analyses were performed using the R programming language version 2.8.0 [19].

## Results

**Figure 1:**
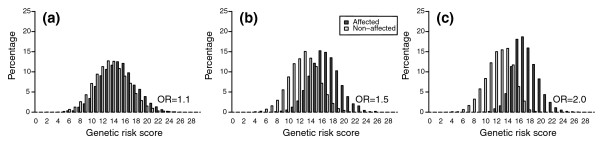
**Distribution of genetic risk scores in affected and non-affected individuals**. Genetic risk scores are based on 50 genetic risk variants. **(a-c) **Each risk variant has an OR of 1.1 (a), 1.5 (b) and 2 (c). Disease risk is 30%.

Figure 1 shows the distribution of genetic risk scores in affected and non-affected individuals for different ORs of the variants included. In our simulation study that included 50 genetic variants, the theoretical range of the risk score was 0 to 100, but the observed range was 2 to 32 with a median of 15 risk alleles. The AUC for the risk scores was 0.62 when the OR of each included variant was 1.1, 0.86 when the OR was 1.5, and 0.94 when the OR was 2.

**Figure 2:**
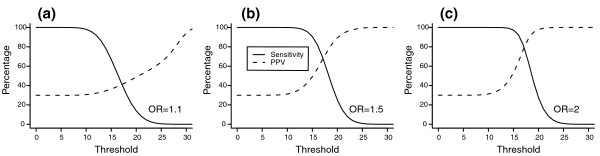
**Sensitivity and positive predictive value (PPV) for different thresholds**. High-risk group is defined as all individuals with a genetic risk score equal to or higher than the chosen threshold. Genetic risk scores are based on 50 genetic risk variants. **(a-c) **The OR indicates the value of the odds ratio for each risk variant: 1.1 (a), 1.5 (b) and 2 (c). Disease risk is 30%.

Figure 2 shows the sensitivity and PPV for all possible thresholds of the genetic risk scores. When a higher threshold is used, the population at high risk has a higher risk (higher PPV), but this will identify a smaller percentage of the affected individuals (lower sensitivity). Comparison of the graphs (Figure 2a-c) shows that for thresholds within the observed range of genetic risk scores, sensitivity and PPV were higher for higher ORs of the individual polymorphisms. When, for example, 15 was taken as the threshold risk score, the sensitivity was 67%, 91% and 97% and the PPV was 36%, 49% and 53% when the OR of each genetic variant was 1.1, 1.5 and 2, respectively. Using a higher threshold increased the specificity but decreased the NPV (Additional file 2).

**Figure 3:**
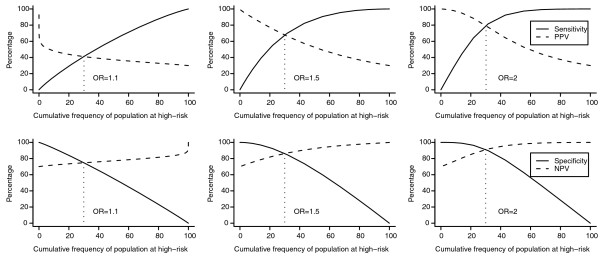
**Sensitivity, specificity, and positive and negative predictive value (PPV, NPV) for different frequencies of the population at high risk**. The frequency of the population at high risk is defined as the proportion of individuals with a number of risk alleles equal to or higher than the chosen threshold. The graphs in the upper row show the sensitivity and PPV for all possible risk thresholds, and the graphs in the lower row the specificity and NPV. Genetic risk scores are based on 50 genetic risk variants. The OR indicates the value of the odds ratio for each risk variant. Disease risk is 30%.

Figure 3 shows the relationship between the frequency of the high-risk group and the sensitivity, PPV, specificity and NPV. With increasing frequency of the population at high risk, sensitivity increased while PPV decreased; and specificity decreased while NPV increased. Note that because higher thresholds yield smaller high-risk categories, the lines depicting sensitivity and PPV show opposite trends in Figures 2 and 3. Figure 3 shows that when, for example, the top 10% of the risk score distribution was considered the high-risk group, sensitivity was 14% when the OR of each genetic variants was 1.1, indicating that most of the affected individuals were not detected. Sensitivity increased to 25% and 28% when OR was 1.5 and 2, showing that sensitivity did not markedly increase with increasing OR. The corresponding PPV values were 49%, 89% and 98%, indicating that PPV increased substantially with increasing OR. Figure 3 shows that the lines cross when the frequency of the high-risk group is equal to 30%, that is, the frequency of disease in the total population. The pattern remained the same when we repeated the analyses for a disease risk of 10% (Additional file 3).

**Figure 4:**
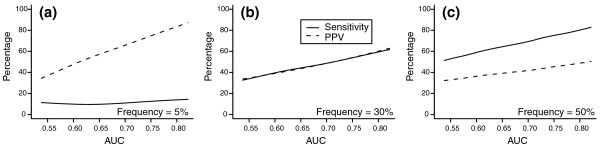
**Sensitivity and positive predictive value (PPV) when the frequency of the high-risk group is lower than, equal to or higher than the disease risk**. The frequency of the high-risk group is defined as the proportion of individuals with a number of risk alleles equal to or higher than the chosen threshold. **(a-c) **High-risk groups have a frequency of 5% (a), 30% (b) and 50% (c). Five to 600 variants are included in the genetic risk models to obtain an increase in the AUC. Each risk variant has a frequency of 30% and OR of 1.1. Disease risk is 30%.

Increasing the OR of all variants included in the genetic risk score also increases the AUC of the risk score. Figure 4 shows the impact of increasing AUC on sensitivity and PPV for high-risk groups that were of lower, equal or higher frequency than the disease frequency in the population. The AUC ranged from 0.51 to 0.82. When the frequency of the high-risk group was lower than disease frequency, PPV markedly increased with increasing AUC, but sensitivity remained low even for high AUC because, by definition, the high-risk group was rarer than the disease (Figure 4a). When the frequency of the high-risk group was higher than the disease risk, sensitivity reached around 80% but PPV remained below 50% when AUC was 0.82 (Figure 4c). Only when the size of the high-risk group was equal to the disease risk in the population were sensitivity and PPV approximately equal and both increased with the increase in AUC (Figure 4b). However, when AUC was 0.82 both sensitivity and PPV were only slightly higher than 60%. Similarly, specificity and NPV were equal only when high-risk groups had a frequency equal to disease risk (data not shown).

**Figure 5:**
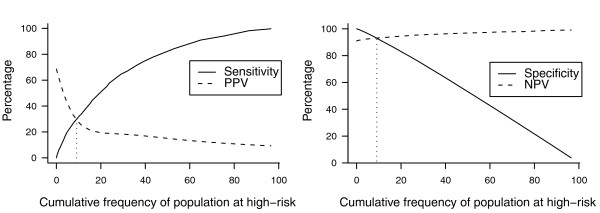
**Sensitivity, specificity, and positive and negative predictive value (PPV, NPV), for age-related macular degeneration simulation**. Predicted risks of age-related macular degeneration are obtained using logistic regression analysis based on six genetic variants entered as categorical variables. The frequency of the population at high-risk is defined as the proportion of individuals with predicted risks equal to or higher than the chosen risk threshold. The genotypic odds ratios and frequencies were obtained from the paper by Seddon *et al*. [16]. Disease risk is 9%.

Finally, we examined the same associations using simulated data based on published ORs and frequencies for six known AMD genetic risk factors. The range of predicted risks was 0.2% to 62% (Additional file 4, which shows the distribution of predicted risks in individuals with and without AMD) and the AUC was 0.76 (95% confidence interval 0.74 to 0.78). We observed the same impact of the relative magnitude of the size of the high-risk groups and disease risk on the sensitivity, specificity, PPV and NPV as in our main simulation study (Figure 5).

## Discussion

This study investigated the relationships between sensitivity, PPV, prevalence of risk group and disease prevalence when genetic risk scores, as opposed to single risk variants, are used for risk stratification. A major finding from this analysis is that when the frequency of the high-risk group approximates the disease frequency, both sensitivity and PPV increase with higher AUC. At all other frequencies of the high-risk group, higher AUC will increase either sensitivity or PPV. Selecting the optimal cut-off threshold will consequently be a trade-off between higher sensitivity at the price of lower PPV, or vice versa.

While the relationship between the number of individuals carrying a certain genetic risk factor and the risk of disease in the population was shown to influence the screening performance for a single marker [15], we have proven this is also true for a genetic test composed of multiple genetic risk factors. Furthermore, we extended the analyses to the context of the overall model performance, and looked at the influence of the discriminatory ability of a genetic model on screening parameters for risk groups with a frequency lower than, equal to or higher than the disease risk.

Genetic tests are usually assessed in terms of their ability to distinguish risk groups with large differences in risk. Nevertheless, it has been shown that large relative risks are not sufficient to demonstrate the model's clinical validity and utility [20-22]. Measures like sensitivity, specificity, PPV and NPV are needed to determine the clinical utility of the test [22]. While sensitivity and specificity are not affected by the incidence of disease because they are characteristics of the test, PPV and NPV strongly depend on disease risk. However, even for rare diseases, risk groups with a high PPV may be selected. Kraft *et al*. [22] used the example of prostate cancer 5-year risk prediction to illustrate this. They show that 60-year-old men with nine or more risk alleles and a positive family history for prostate cancer, which represent 1% of the population, have a risk of 30% to develop prostate cancer over the next 5 years. The incidence of disease in the population of 60-year-old men is about 2%. Thus, the size of the group at high risk was smaller than disease risk. We show that in addition to a smaller size of the high risk group and high OR for the risk factors, a high AUC is needed to obtain a high PPV. In a recent study the AUC of a genetic score of 33 SNPs and family history of prostate cancer was estimated at 0.64 [23]. A higher AUC is needed to select a risk group with bigger PPV, especially if the high risk group is targeted for invasive interventions.

The observation that the sensitivity and PPV are equal when the frequency of the high-risk group equals the frequency of disease in the population holds across different settings. First, this relationship holds irrespective of whether the disease risk refers to the lifetime risk, a cumulative incidence over certain time period or the disease prevalence. Evidently, if we consider, for example, lifetime risks instead of 10-year risks, the frequency of the high-risk group for which the sensitivity and PPV are equal will be larger, because lifetime risks by definition are higher than 10-year risks. Then for the same AUC values, these larger high-risk groups will have higher sensitivity and PPV. However, prediction models that consider longer time periods generally have lower AUC, implying that combinations of higher sensitivity and PPV may not be observed. Put differently, lifetime risk models with lower AUC may yield the same sensitivity/PPV combination as 10-year risk models with higher AUC, but the value of using a model with low AUC may become questionable.

Second, the relationship also holds irrespective of how the risks are calculated. There are several ways in which genetic risks can be expressed. One is to use a simple genetic risk score based on the number of risk alleles carried. This approach, which we used in our analyses, assumes that each allele has the same effect on the risk of disease [24,25]. Another option is to calculate a weighted risk score, which is a genetic risk score where the risk alleles are weighted for their effect on disease risk [14]. Besides constructing risk scores, one can also directly derive predicted risks from multivariate logistic regression analyses with genetic variants entered as continuous or categorical variables. Results presented in this study are applicable to simple count scores and more complex weighted risk scores, such as predicted risks, as emphasized by the simulation of AMD risk prediction, since in this study we have evaluated cut-off values that simply dichotomize the risk. Nevertheless, it should be pointed out that different approaches will likely yield different AUC values.

Third, the relationship also holds for risk models in general, that is, including other non-genetic risk models, such as the Framingham risk score for prediction of cardiovascular disease. Basically the relationship is valid for any continuous variable that is dichotomized to create risk groups, such as blood pressure, cholesterol or triglyceride level. This is also true for risk models that include together novel biomarkers and established risk factors, a topic that has recently attracted a lot of research [26,27].

When risk models are used to target interventions to high-risk subgroups, these subgroups are defined by choosing cut-off values for the predicted risks. The cut-off corresponding to a frequency of the high-risk group equal to the disease frequency optimizes both the sensitivity and the PPV, but is not necessarily optimal. Cut-off values are chosen on the basis of cost-benefit analyses, balancing the harms and benefits of false positive and false negative classifications of risk. The cut-off defining a risk group with a frequency equal to disease frequency is optimal only when the harm and benefit have equal weights. Selection of optimal cut-off based on a decision-analytic approach is a complex process that requires detailed input information of measures like sensitivity, specificity, PPV, NPV and related costs. For example, a recent study reported the effect of family history and 14 SNPs on the cost-effectiveness of chemoprevention with finasteride for prostate cancer [28]. The results show that genetic testing may marginally improve the cost-effectiveness of chemoprevention in individuals with more risk alleles, especially in men with a positive family history. However, no optimal cut-off number of risk alleles was determined and the cost-effectiveness varied significantly with small changes of the model parameters. Our analyses do show, however, that when AUC is low to moderate, selecting a subgroup with a substantially increased risk (that is, high PPV) will include only a small percentage of all people who will develop the disease (that is, low sensitivity). Obviously, the predictive ability is the fundamental prerequisite of a test, but what level of predictive ability is needed varies between applications.

Our observations have implications for health care applications of genetic testing, but also for the direct-to-consumer offer of personal genome tests via the internet. For health care applications that need high PPV, such as targeting invasive interventions to people at the highest risk, a low AUC means that only a small proportion of this group will be identified. For applications that need high sensitivity, such as screening programs, the interventions will be given to a very large part of the population, mostly to people who will not develop the disease. And finally, low AUC means for personal genome testing that most people who will develop the disease will not be identified as having high risks.

## Conclusions

Anticipating the advances in this field, it is essential to develop more rigorous approaches to evaluate the clinical usefulness of risk models [29,30]. We have shown that when a threshold for genetic risk is used for selection of individuals at high risk to develop disease in the future, sensitivity, specificity and PPV of the test are strongly influenced by the relative magnitude of the size of the high-risk group and the disease risk in the population. In addition, selection of high-risk groups with clinically useful combinations of sensitivity and PPV is only possible when the AUC values are higher.

## Abbreviations

AMD: age-related macular degeneration; AUC: area under the receiver operating characteristic curve; NPV: negative predictive value; OR: odds ratio; PPV: positive predictive value; SNP: single-nucleotide polymorphism.

## Competing interests

The authors declare that they have no competing interests.

## Authors' contributions

ACJWJ and RM conceived the study and drafted the manuscript. RM performed the statistical analysis. RM and MJK participated in the design and helped to draft the manuscript. All authors read and approved the final manuscript.

## Supplementary Material

Additional file 1 **Supplementary tables and supplementary figure legends**. A table listing genotype ORs and genotypic frequencies of the markers included in the AMD simulation and figure legends for Additional files 2 to 4. Click here for file

Additional file 2 **Supplementary Figure S1**. A figure showing the change in specificity and NPV for different thresholds. Click here for file

Additional file 3 **Supplementary Figure S2**. A figure showing the sensitivity, specificity, PPV and NPV for different frequencies of the population at high risk. Click here for file

Additional file 4 **Supplementary Figure S3**. A file showing the distribution of predicted risks in individuals with and without AMD. Click here for file
