# Supplementary material for: Predictive genetic testing for the identification of high-risk groups: a simulation study on the impact of predictive ability
Source: Genome Med. 2011 Jul 28;3(7):51. doi: 10.1186/gm267 (PMC3221548; doi:10.1186/gm267)
Supplement: Additional file 1 — Supplementary tables and supplementary figure legends. A table listing genotype ORs and genotypic frequencies of the markers included in the AMD simulation and figure legends for Additional files 2 to 4. [file gm267-S1.DOC]

**Web Only Data**

**SUPPLEMENTARY TABLE 1**

**Genotypic parameters for the age-related macular degeneration simulation.**

OR, odds ratio; SNP, single nucleotide polymorphisms.

^1^Values are for heterozygous and homozygous carriers of the associated alleles or for the heterozygous and homozygous carriers combined.

**SUPPLEMENTARY FIGURES**

**Figure S1. Specificity and negative predictive value (NPV) for different thresholds.**

: High-risk group is defined as all individuals with a genetic risk score equal or higher than the chosen threshold. Genetic risk scores are based on 50 genetic risk variants. The OR indicates the value of the odds ratio for each risk variant. Disease risk is 30%.

**Figure S2. Sensitivity, specificity, positive and negative predictive value (PPV, NPV) for different frequencies of the population at high-risk.**

The frequency of the population at high-risk is defined as the proportion of individuals with a number of risk alleles equal or higher than the chosen threshold. The graphs in the upper row show the sensitivity and PPV for all possible risk thresholds, and the graphs in the lower row the specificity and NPV. Genetic risk scores are based on 50 genetic risk variants. The OR indicates the value of the odds ratio for each risk variant. Disease risk is 10%.

**Figure S3.** **Distribution of predicted risks in individuals with and without age-related macular degeneration.**

Predicted risks are obtained from logistic regression analysis with age-related macular degeneration as outcome variable and 6 genetic risk variants entered as categorical predictor variables. We used the same genotypic odds ratios and frequencies as in the paper by Seddon et al.^16^ Disease risk is 9%.

**Supplementary Table 1.**

| **Gene** | **SNP** | **Associated allele** | **OR^1^** | **Frequency, %^1^** |
| --- | --- | --- | --- | --- |
| *CFH* | rs1061170 | C | 2.1/4.1 | 44.6/24.0 |
| *CFH* | rs1410996 | C | 3.1/7.2 | 40.4/46.0 |
| *LOC387715* | rs10490924 | T | 2.8/6.2 | 38.2/9.3 |
| *C2* | rs9332739 | C | 0.3 | 7.9 |
| *CFB* | rs641153 | T | 0.6 | 12.3 |
| *C3* | rs2230199 | G | 1.5/2.2 | 39.1/5.1 |
