# Supplementary figures and images for: Predictive genetic testing for the identification of high-risk groups: a simulation study on the impact of predictive ability
Source: Genome Med. 2011 Jul 28;3(7):51. doi: 10.1186/gm267 (PMC3221548; doi:10.1186/gm267)

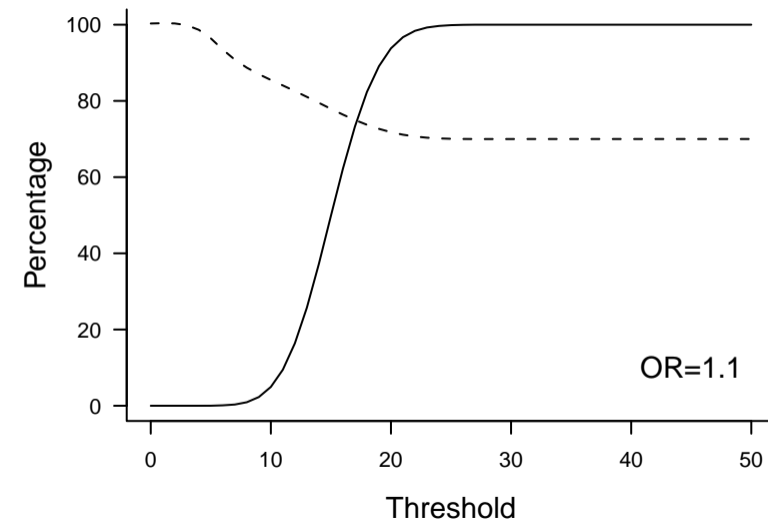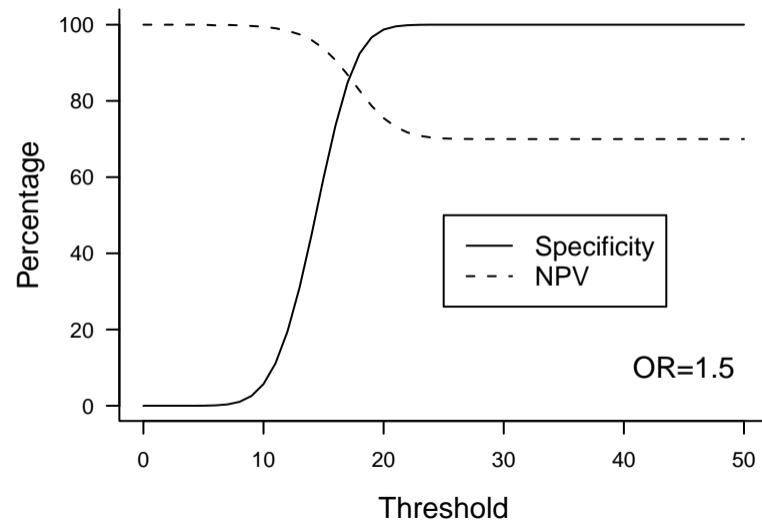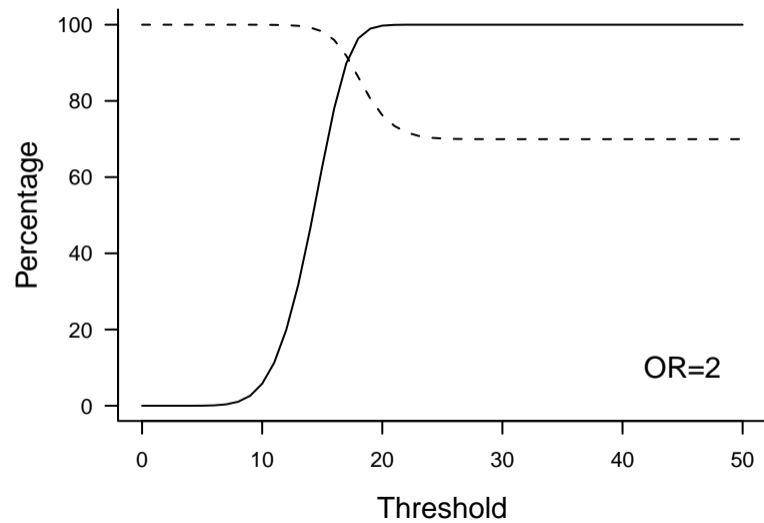

Supplement: Additional file 2 — Supplementary Figure S1. A figure showing the change in specificity and NPV for different thresholds. [file gm267-S2.PDF]

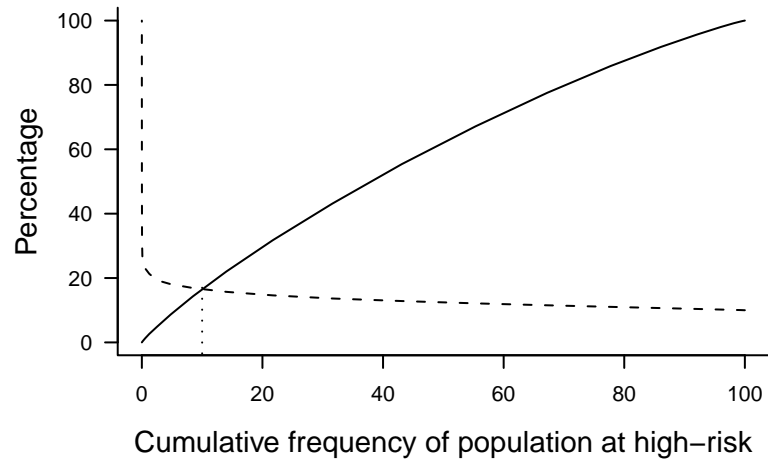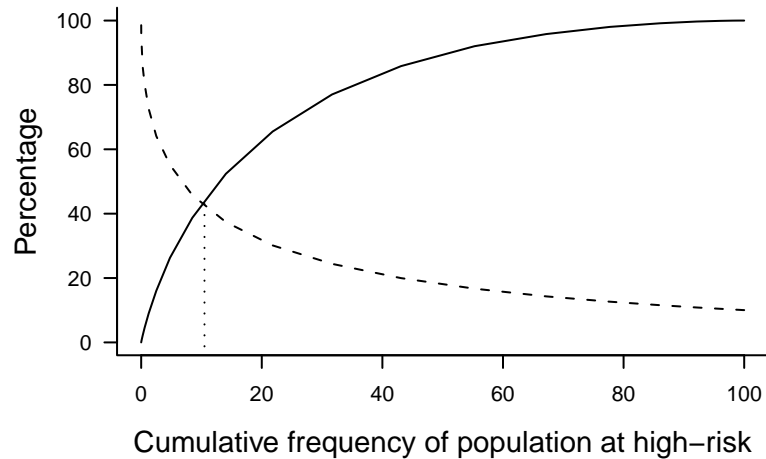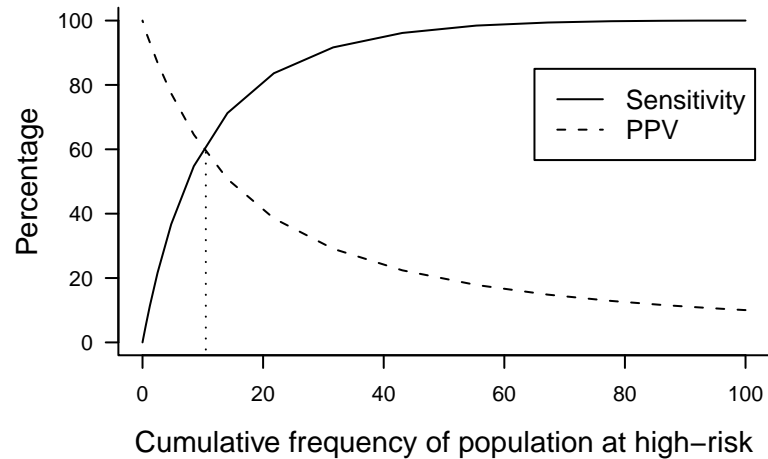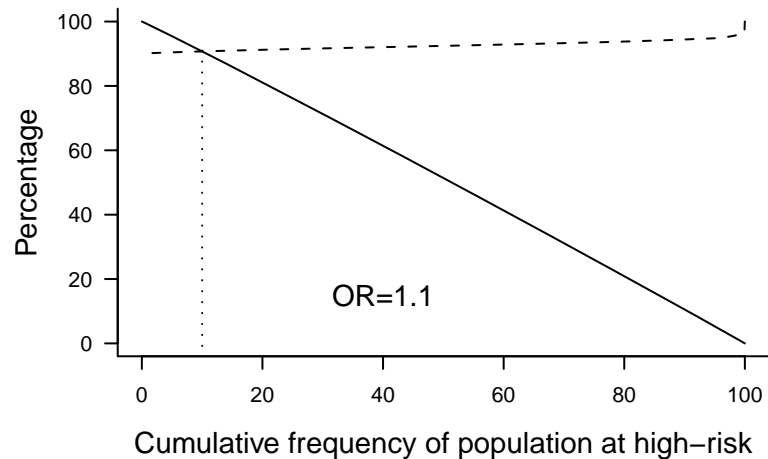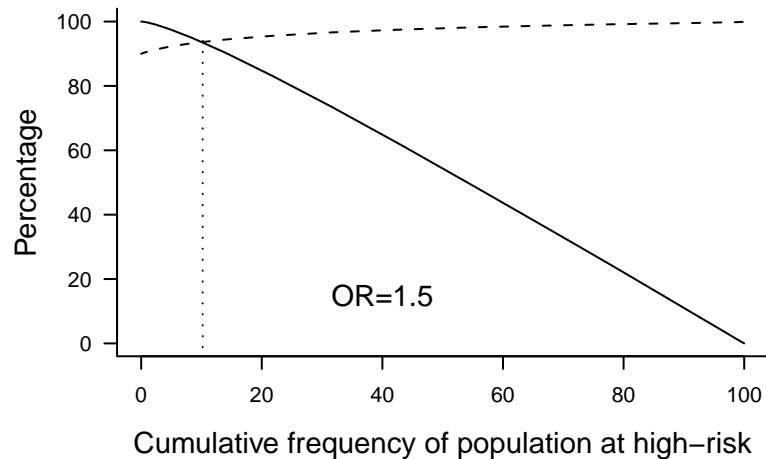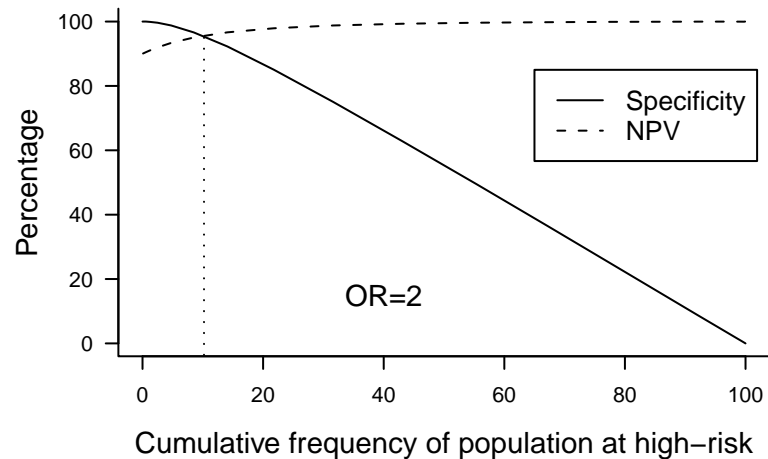

Supplement: Additional file 3 — Supplementary Figure S2. A figure showing the sensitivity, specificity, PPV and NPV for different frequencies of the population at high risk. [file gm267-S3.PDF]

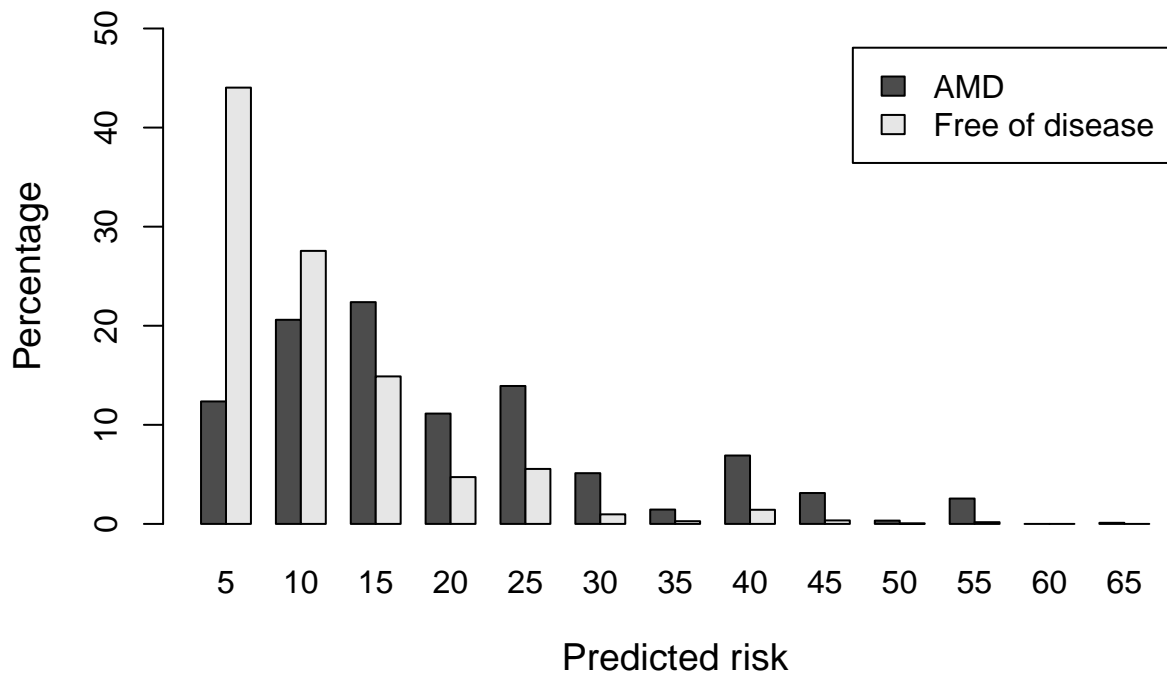

Supplement: Additional file 4 — Supplementary Figure S3. A file showing the distribution of predicted risks in individuals with and without AMD. [file gm267-S4.PDF]
